# Local Regulation of Striatal Dopamine Release Shifts from Predominantly Cholinergic in Mice to GABAergic in Macaques

**DOI:** 10.1523/JNEUROSCI.1692-24.2025

**Published:** 2025-01-21

**Authors:** Jung Hoon Shin, Hannah C. Goldbach, Dennis A. Burke, Michael E. Authement, Evan S. Swanson, Miriam E. Bocarsly, Sean Hernandez, Han B. Kwon, Sydney E. Cerveny, Jacqueline B. Mehr, Anya S. Plotnikova, Arya Mohanty, Alexander C. Cummins, Kenneth A. Pelkey, Chris J. McBain, Zayd M. Khaliq, Mark A. G. Eldridge, Bruno B. Averbeck, Veronica A. Alvarez

**Affiliations:** ^1^Laboratory on Neurobiology of Compulsive Behaviors, National Institute on Alcohol Abuse and Alcoholism, NIH, Bethesda, Maryland 20892; ^2^Comparative Brain Physiology Consortium, Center on Compulsive Behaviors, National Institutes of Health, Bethesda, Maryland 20892; ^3^Laboratory on Neuronal Circuits and Behavior, National Institute of Mental Health, NIH, Bethesda, Maryland 20892; ^4^Laboratory of Neuropsychology, National Institute of Mental Health, National Institutes of Health, Bethesda, Maryland 20892; ^5^Section on Cellular and Synaptic Physiology, Eunice Kennedy Shriver National Institute of Child Health and Human Development (NICHD), Bethesda, Maryland 20892; ^6^Cellular Neurophysiology Section, National Institute of Neurological Disorders and Stroke, National Institutes of Health, Bethesda, Maryland 20892; ^7^Aligning Science Across Parkinson’s (ASAP) Collaborative Research Network, Chevy Chase, Maryland 20815

**Keywords:** acetylcholine, caudate putamen, dopamine sensor, nicotinic receptors, primates, voltammetry

## Abstract

Dopamine critically regulates neuronal excitability and promotes synaptic plasticity in the striatum, thereby shaping network connectivity and influencing behavior. These functions establish dopamine as a key neuromodulator, whose release properties have been well studied in rodents but remain understudied in nonhuman primates. This study aims to close this gap by investigating the properties of dopamine release in macaque striatum and comparing/contrasting them to better-characterized mouse striatum, using ex vivo brain slices from male and female animals. Using combined electrochemical techniques and photometry with fluorescent dopamine sensors, we found that evoked dopamine signals have smaller amplitudes in macaques compared with those in mice. Interestingly, cholinergic-dependent dopamine release, which accounts for two-thirds of evoked dopamine release in mouse slices, is significantly reduced in macaques, providing a potential mechanistic underpinning for the observed species difference. In macaques, only nicotinic receptors with alpha-6 subunits contribute to evoked dopamine release, whereas in mice, both alpha-6 and non-alpha6-containing receptors are involved. We also identified robust potentiation of dopamine release in both species when GABA_A_ and GABA_B_ receptors were blocked. This potentiation was stronger in macaques, with an average increase of 50%, compared with 15% in mice. Together, these results suggest that dopamine release in macaque is under stronger GABA-mediated inhibition and that weaker cholinergic-mediated dopamine release may account for the smaller amplitude of evoked dopamine signals in macaque slices.

## Significance Statement

Dopamine plays a vital role in striatal function, influencing both essential physiological processes and the development of debilitating brain disorders. Most of our current understanding of dopamine release and regulation stems from rodent studies. Investigating the similarities and differences between rodent and primate dopamine systems will improve translatability of findings from rodents to humans. This comparative study identified differences in the local regulation of dopamine release within the striatum of macaques and mice. The modulation by acetylcholine was weaker while the modulation by GABA was stronger in macaques.

## Introduction

Dopamine projections to the striatum underlie many important functions, including movement control and reinforcement learning (Schultz, 2002). Loss of dopamine innervation to the dorsal striatum leads to Parkinson's disease, and excessive dopamine in the same region can lead to the positive symptoms of schizophrenia (Gerfen and Engber, 1992; Chen et al., 2024). Furthermore, the actions of dopamine in the striatum are implicated in substance use disorder and other compulsive behaviors (Nestler, 2005; Jalal et al., 2023). Understanding dopamine release regulation in the striatum will provide mechanistic knowledge of these physiological and pathological states.

Much of what we know about dopamine release in the striatum, particularly at the synaptic level, comes from work in rodents (Rice et al., 2011; Gantz et al., 2018; Ozcete et al., 2024). Mice are an important model system because of the availability of genetically modified lines which, in combination with virally delivered constructs, provide detailed and precise control over function at the molecular, synaptic, and cellular levels. While mice offer unique advantages, there are important differences in forebrain circuits between mice and primates which make it unlikely that dopamine release and its regulation would be the same across the species. These differences encompass the overall scale of the forebrain and the existence of several structures in primates that are not found in mice, such as the granular lateral prefrontal cortex, the internal capsule, and a well-defined globus pallidus internal segment (https://scalablebrainatlas.incf.org/macaque/PHT00; Bezgin et al., 2009; Bakker et al., 2015). Relevant for dopamine regulation, there are differences between rodents and primates in the organization of the reciprocal interaction between the striatum and midbrain dopamine neurons (Joel and Weiner, 2000). For example, in rodents the projections from the motor and associative striatum to the dopamine neurons are largely topographically segregated into a lateral-medial gradient in the midbrain while in primates caudate and putamen projections overlap in the midbrain, suggesting convergence. Similarly, the dopamine neuron populations that project to lateral and medial striatal subregions are topographically segregated in rodents while in primates they form interdigitated clusters. The dopamine innervation of the cortex is also expanded in primates relative to rats and shows differences in the laminar pattern of projections (Berger et al., 1991). This suggests that dopamine release and regulation may be different between rodents and primates. In fact, the comparison of evoked dopamine release in the striatum between guinea pigs and marmosets, a new-world primate, has shown increased dopamine release in the marmoset relative to the guinea pig (Cragg et al., 2000). However, a study comparing mice and macaques found the opposite, with macaques showing less striatal dopamine release than mice (Calipari et al., 2012). Understanding the comparative similarities and differences between rodent and primate dopamine systems can lead to a better understanding of the ways in which findings in rodents translates, ultimately, to humans.

The striatum, a nucleus dominated by GABA-expressing neurons, relies on inhibition and disinhibition as critical features of the local circuitry that drive computation and information processing. Among the multiple interspecies differences between rodents and primates, an expansion of inhibitory cell types has been shown in primate cortex and striatum (Boldog et al., 2018; Krienen et al., 2020; Corrigan et al., 2024), raising the possibility that inhibition and disinhibition are different in primate brains. In mice, GABA receptors are known to influence dopamine release through multiple mechanisms, including by sensing striatal GABAergic tone and GABA coreleased from dopamine axons themselves (Gruen et al., 1992; Smolders et al., 1995; Pitman et al., 2014; Lopes et al., 2019; Kramer et al., 2020; Holly et al., 2021; Patel et al., 2024). Based on the reported difference between species in proportion of GABA-expressing neurons, we speculated that GABA modulation of dopamine transmission might be changed in macaques compared with mice.

In addition to GABA, striatal dopamine release is also modulated by acetylcholine. Acetylcholine regulates dopamine release via nicotinic receptors expressed on dopamine axons within the striatum (Jones et al., 2001; Rice et al., 2011; Kramer et al., 2022). This nicotinic-mediated regulation of dopamine release may serve specific functional roles that are distinct from release evoked by the firing of dopamine neurons initiated at the soma (Mohebi et al., 2019; Adrover et al., 2020; Kramer et al., 2022; Liu et al., 2022). For example, whereas spiking activity in dopamine neuron cell bodies correlates with reward prediction errors under many conditions (Fritsche et al., 2024; Schultz, 2024), regulation of terminal release within the striatum might correlate with state value and/or salience (Hamid et al., 2016; Mohebi et al., 2019; Jeong et al., 2022; Gershman et al., 2024).

In the present study, we compared dopamine release capacity and regulation between mouse and macaque ex vivo brain slices. Earlier work on this topic relied on fast-scan cyclic voltammetry (FSCV), an electrochemical technique. Here, in addition to voltammetry, we further approached this question of interspecies comparison with optical methods using virally expressed genetically encoded fluorescent dopamine sensors, dLight1.3b and GRAB-DA1m. Furthermore, we examined the regulation of dopamine release by two neurotransmitters known to influence release in rodents: acetylcholine and GABA. In agreement with previous work, we found smaller evoked dopamine signals in macaques compared with mice and the existence of a ventrodorsal gradient in both species, with larger dopamine release in the putamen/dorsolateral striatum than the ventral striatum/nucleus accumbens. Extending from the previous report, this study identifies that the cholinergic contribution to the evoked dopamine release is smaller in macaques compared with mice, possibly explaining the smaller overall amplitude in macaques. Indeed, removing the cholinergic contribution led to evoked dopamine levels that were comparable between mice and macaques. Finally, dopamine regulation by GABA was stronger in macaque than mouse brain slices. Blocking both ionotropic and metabotropic GABA receptors produced a stronger potentiation of dopamine release in the macaque striatum compared with the mouse striatum. Altogether, this comparative study offers evidence of evolutionary divergence on dopamine release regulation between mice and macaques.

## Materials and Methods

All measurements presented in the study were performed in ex vivo brain slices from rhesus macaques and C57BL/6J mice.

### Animals

All experiments were performed in accordance with the ILAR Guide for the Care and Use of Laboratory Animals and were conducted under an Animal Study Protocol approved by the ACUC at the National Institute of Mental Health and National Institute on Alcohol Abuse and Alcoholism. All procedures adhered to the applicable Federal and local laws, regulations, and standards, including the Animal Welfare Act and Regulations and Public Health Service policy (PHS2002). All animals were housed on a 12 h light/dark cycle (06:30 to 18:30 light) with food and water *ad libitum*. Macaque tissue was obtained from 17 adult rhesus macaques (*Macaca mulatta*, 7 male and 10 female; age 6–17 years, average age of 11.1 years), as part of the Comparative Brain Physiology Consortium at the NIH. Mouse tissue was obtained from C57BL/6J mice (31 male and 14 female; age 9–58 weeks, average age of 24.9 weeks).

### Stereotaxic vector injection

For macaques, animals were sedated with ketamine/midazolam (ketamine 5–15 mg/kg, midazolam 0.05–0.3 mg/kg) and maintained on isoflurane and placed in a stereotaxic frame. Surgeries were performed under aseptic conditions in a fully equipped operating suite. Viral injections were unilateral and targeted either the right or left striatum using stereotaxic coordinates derived from MRI for enhanced accuracy (Fredericks et al., 2020). Between 9 and 12 locations (2–2.5 mm apart) were injected with 10 μl/site of viral vector solution delivered via a needle using a syringe pump. The vector constructs used were AAV9-hSyn-GRAB-DA1m (2.6 × 10^13^ IU/ml; 113049-AAV9 from Addgene), AAV5-CAG-dLight1.3b (>7 × 10^12^ IU/ml; 125560-AAV5 from Addgene), and AAV5-Syn-dLight1.3b (2.3 × 10^13^ IU/ml; Vigene). Brain extraction took place 5–7 weeks after viral injection. One female (age 12.1 years) and three male macaque (ages 8.7, 10.6, and 10.6 years) contributed to these experiments. For mice, stereotaxic injections were conducted as described previously (Adrover et al., 2014). Briefly, mice were anesthetized by inhalation of isoflurane-oxygen mixture and placed in a stereotaxic frame (David Kopf). Bilateral injections into the dorsal striatum (AP: 1.1, ML: ± 1.4, DV: −3.25) were made via a glass electrode (250 nl/side) using injector Nanoject III (Drummond Scientific). Vectors used were AAV9-hSyn-GRAB-DA1m (2.6 × 10^13^ IU/ml; 113049-AAV9 from Addgene) or AAV5-syn-dLight1.3b (2.3 × 10^13^ IU/ml; Vigene). All stereotaxic coordinates were from bregma (in mm) according to the mouse atlas by Franklin and Paxinos (2008). Brain extraction took place 5–9 weeks after viral injections for most of animals and 18–41 weeks for three mice.

### Brain extraction and slice preparation

For macaque brain extraction, the animals were sedated with ketamine/midazolam (ketamine 5–15 mg/kg, midazolam 0.05–0.3 mg/kg) and maintained on isoflurane. A deep level of anesthesia was verified by an absence of response to toe-pinch and absence of response to corneal reflex. Prior to brain removal and blocking, macaques were transcardially perfused with ice-cold sucrose-substituted artificial cerebrospinal fluid (SSACSF) containing the following (in mM): 90 sucrose, 80 NaCl, 24 NaHCO_3_, 10 glucose, 3.5 KCl, 1.25 NaH_2_PO_4_, 4.5 MgCl_2_, and 0.5 CaCl_2_ saturated with 95% O_2_ and 5% CO_2_. Macaque brains were further sectioned to tissue blocks containing striatum before thin sections were prepared. For mouse brain extraction, animals were anesthetized with isoflurane and brains quickly removed following decapitation and the same ice-cold SSACSF used. Macaque and mouse coronal brain slices (300 µm for macaques and 240 µm for mice) were prepared using a Leica 1200 S Vibratome in the SSACSF solution saturated with 95% O_2_ and 5% CO_2_. Slices were incubated in a warm (∼32°C) SSACSF solution for 20–30 min and then returned to room temperature until recording. For recording, slices were transferred to a submerged recording chamber and perfused at 2 ml/min with extracellular artificial cerebrospinal fluid (ACSF) containing the following (in mM): 124 NaCl, 26.2 NaHCO_3_, 20 glucose, 2.5 KCl, 1 NaH_2_PO_4_, 2.5 CaCl_2_, 1.3 MgCl_2_, and 0.4 ascorbic acid saturated with 95% O_2_ and 5% CO_2_, which was heated at 32°C using an inline heater (Harvard Apparatus).

### Fast-scan cyclic voltammetry

FSCV was performed in the striatum. Carbon fiber electrodes were prepared with a cylindrical carbon fiber (7 µm in diameter; 150 µm of exposed fiber) inserted into a glass pipette, which was backfilled with 3 M KCl. Before use, the carbon fiber electrodes were conditioned with an 8-ms-long triangular voltage ramp (−0.4 to +1.2 and back to −0.4 V vs Ag/AgCl reference at 400 V/s) delivered every 15 ms. The carbon fiber electrode is inserted diagonally into the slice, with a ∼22° angle to maximize contact between carbon fiber surface and the tissue and minimize the force that pushes down into the tissue. During the recording, the carbon fiber electrodes were held at −0.4 V versus Ag/AgCl, and the same triangular voltage ramp was delivered every 100 ms. For electrical stimulation, a glass pipette filled with ACSF was placed near the tip of the carbon fiber and a rectangular pulse (0.2 ms; SIU91A by Cygnus Technologies) was applied every 2 min. For input–output curves, the current amplitude to the stimulation electrode was adjusted to 5, 10, 15, 20, 40, 60, 80, 100, 150, 200, and 300 µA. Data were collected with a retrofit headstage (CB-7B/EC with 5 MΩ resistor) using a MultiClamp 700B amplifier (Molecular Devices) after low-pass filter at 3 kHz and digitized at 100 kHz using a digital–analog board (NI USB-6229 BNC, National Instruments). Data acquisition and analysis were performed using a custom-written software, VIGOR, in Igor Pro (WaveMetrics) using mafPC (courtesy of MA Xu-Friedman). The current peak amplitudes of the evoked dopamine transients were converted to dopamine concentration according to the calibration using 1–3 µM dopamine.

### Photometry

Dopamine sensors expressed in slices were illuminated by 470 nm LED light (<2% of the maximum output) from a pE-800 (CoolLED) through 40× lens, and the fluorescence light was collected by a photomultiplier tube (R1527P by Hamamatsu) with the anode to cathode voltage of −700 V provided by a PS310 power supply (Stanford Research Systems). The current from the photomultiplier tube was low-pass filtered at 300 Hz and converted to voltage using a SR570 preamplifier (Stanford Research Systems). The voltage signal was digitized at 5 kHz using pClamp10 software (Molecular Devices), which was then converted to %*ΔF*/*F* off-line using Igor Pro.

### Statistical analysis

Statistical analysis was performed with Prism (GraphPad). The following tests were used, unless specified: for pairwise comparisons, we use two-tailed *t* test; for multiple group comparisons, we used ANOVA, with repeated measures and with or without mixed-effects models as appropriate. One-sample *t* test was used when comparing to baseline control. Sidak multiple-comparison test was used following ANOVAs when appropriate. The number of experiments, *n*, was expressed as the number of slices/the number of animals, and it is stated in the figure legends as well as in the text if there is no figure.

### Drugs and chemicals

Dihydro-β-erythroidine hydrobromide (DHβE) and CGP55845 hydrochloride were purchased from Tocris, α-conotoxin-PIA from Alomone Labs, SR95531 (gabazine) from Hello Bio, and all other chemicals from Sigma-Aldrich.

## Results

In these experiments, we compared electrically evoked dopamine release across different subregions of the striatum, and between mice and macaques. Evoked dopamine was measured in ex vivo brain slices using both FSCV and, in some cases, the dopamine-sensitive fluorescent reporters dLight1.3b and GRAB-DA1m. We further used pharmacological methods to examine the relative contributions of cholinergic and GABA-mediated mechanisms to the evoked dopamine release.

**Figure 1.**
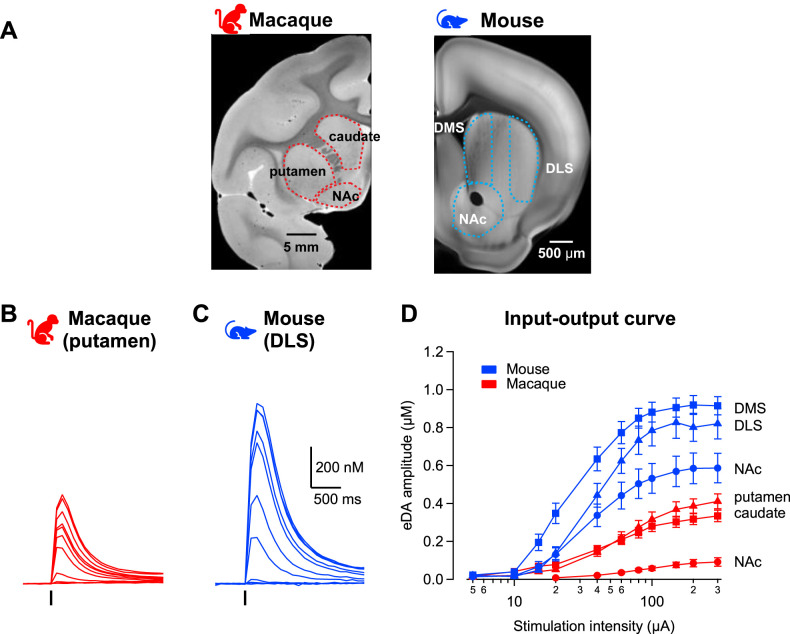
Dopamine signals across the striatum are smaller in macaques compared with mice. ***A***, Coronal section images from macaque (left) and mouse (right) delineating the striatal subregions recorded. Images adapted from Scalable Brain Atlas. NAc, nucleus accumbens; DMS, dorsomedial striatum; DLS, dorsolateral striatum. ***B***, ***C***, Superimposed representative dopamine transients evoked by a single-pulse electrical stimulation of increasing intensity (black tick) from (***B***) the putamen of monkey or (***C***) the DLS of mouse. ***D***, The input–output curves show mean peak amplitude of dopamine signals evoked at each stimulation intensity in the mouse (blue) and macaque (red) striatal subregions (*n* = 33, 23, 31 slices/12, 6, 4 mice; *n* = 23, 31, 16 slices/19, 10, 5 macaques). Symbols and lines are mean ± SEM.

Coronal slices were taken from rhesus macaques (*n* = 17 animals; 7 males/10 females) and mice (*n* = 45 animals; 31 males/14 females). Recordings were carried out in specific subregions of the striatum. In the macaque, subregions were identified by their location relative to the internal capsule. In mice, they were identified by their gross anatomical position (Fig. 1*A*). In the monkey we identified the caudate, putamen, and nucleus accumbens (NAc); in the mouse we recorded from the dorsomedial (DMS), the dorsolateral striatum (DLS), and the NAc (Heilbronner et al., 2016). NAc recordings were made from core and shell areas but data was not segregated due to smaller sample size. All experimental protocols were the same across species and recordings were carried out on the same slice rigs using the same equipment and solutions.

We first examined electrically evoked dopamine release with voltammetry using a series of stimulation currents. Across both species and all striatal subregions, increased stimulation intensity led to increased peak concentration of evoked dopamine signals (Fig. 1*B–D*; three-way repeated-measures ANOVA main effect of stimulation: *F*_(7,1066)_ = 220.1, *p* < 0.001), but with significant differences across striatal subregions (Fig. 1*D*; main effect of area: *F*_(2,148)_ = 9.2, *p* < 0.001). In both species, post hoc analyses showed that the NAc had significantly lower peak concentrations of evoked dopamine than the putamen/DLS and caudate/DMS (post hoc Sidak test both *p*s < 0.001). However, the putamen/DLS and the caudate/DMS did not differ statistically within each species (post hoc Sidak test *p*s > 0.07). Though both species exhibited similar subregion differential response profiles (Fig. 1*D*; interaction species × area: *F*_(2,148)_ = 2.0, *p* = 0.138), evoked dopamine release was lower across all subregions in monkeys compared with that in mice (main effect of species: *F*_(1,148)_ = 105.9, *p* < 0.001). Thus, macaques showed overall lower peak concentrations of evoked dopamine release (400 vs ~800 nM), and dopamine levels were overall lower in the NAc in both species (0.09 ± 0.02 µM in macaques vs 0.59 ± 0.08 µM in mice).

**Figure 2.**
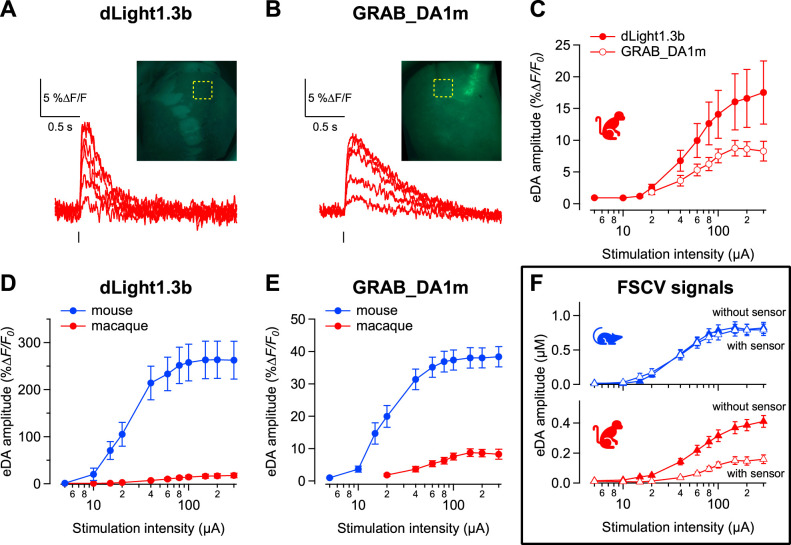
Dopamine fluorescent sensors confirms smaller magnitude of evoked dopamine signals in macaques. ***A***, ***B***, Representative photometry traces obtained from macaque brain slices expressing the fluorescent dopamine sensors dLight1.3b (***A***) and GRAB-DA1m (***B***). Dopamine signals were evoked by increasing stimulation intensities. Top right corner shows fluorescence images of brain sections from macaque caudate and putamen. ***C***, Input–output curves of photometry signals measured in macaque brain sections expressing dLight1.3b (filled symbols, *n* = 14 slices/3 macaques) and GRAB-DA1m (open symbols, *n* = 11 slices/ 2 macaques). ***D***, ***E***, Photometry input–output curves macaque striatum (red) and mouse striatum (blue) when using dLight1.3b (14 slices/3 macaques; 16 slices/7 mice) and GRAB-DA1m (slices *n* = 11 slices/2 macaque; 16 slices/6 mice). ***F***, Voltammetry input–output curves from mouse (blue) and macaque (red) dorsal striatum expressing the fluorescent sensors (open symbols) or not expressing the sensors (filled symbols).

Next, to further validate these voltammetry results, we examined evoked dopamine release using genetically encoded dopamine sensors coupled with photometry. These sensors, consisting of modified dopamine receptors, are expressed directly on the striatal cell membrane, allowing for measurements of dopamine concentrations that more accurately reflect extracellular levels sensed by neurons near dopamine axon boutons (Dong et al., 2022). This is in contrast to the carbon fiber electrode used in voltammetry, which is inserted into the tissue and primarily measures dopamine spillover from release sites. Viral vectors expressing the sensors were injected in the putamen/DLS, and brain sections were prepared 6–8 weeks later to ensure adequate levels of sensor expression (Fig. 2*A–C*). Critically, this independent measure of dopamine concentration confirmed that evoked dopamine transients were larger in the mouse than macaque, as measured by both dLight1.3b (Fig. 2*D*; main effect of species *F*_(1,28)_ = 27.9, *p* < 0.001) and GRAB-DA1m (Fig. 2*E*; main effect of species *F_(_*_1,25)_ = 57.2, *p* < 0.001). In some experiments we carried out simultaneous voltammetry and photometry (Fig. 2*F*). This allowed us to ask whether the presence of the dopamine sensor, which binds dopamine, affects dopamine levels measured with voltammetry. Interestingly, we found that the expression of dopamine sensors had an effect on the amplitude of the dopamine response measured with voltammetry in macaques. Across the stimulation intensities tested, the evoked dopamine responses measured with voltammetry in regions expressing the dopamine sensors were smaller (∼ half) than dopamine responses recorded in regions from the same slice but without sensor expression (main effect of sensor expression; Fig. 2*F*; *F*_(1,36)_ = 4.9, *p* = 0.033). This was the case for both sensors, dLight1.3b and GRAB-DA1m. In mice, there were no differences in evoked dopamine levels from regions with or without expression of these same sensors (Fig. 2*F*; no effect of sensor; *F*_(1,32)_ = 0.04, *p* = 0.839). There was, however, no significant interaction of species and sensor at the highest stimulation level (*F*_(1,68)_ = 2.3, *p* = 0.134).

**Figure 3.**
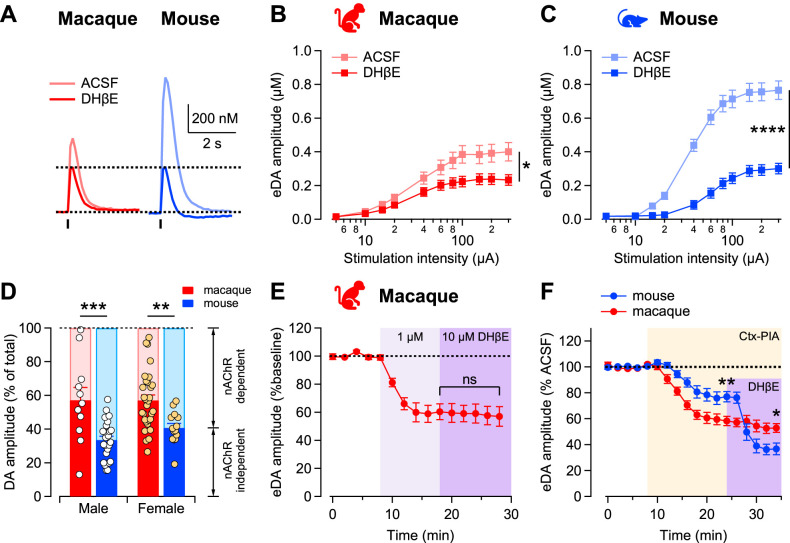
Smaller cholinergic contribution to evoked dopamine signals in macaques compared with mice. ***A***, Representative dopamine transients evoked by single-pulse electrical stimulation (tick) before (pale) and after 1 µM DHβE application (solid) in macaque (red) and mouse (blue). ***B***, ***C***, The input–output curves of dopamine amplitudes with increasing stimulation intensities in macaque (***B***) and mouse (***C***) before (pale) and after (solid) 1 µM DHβE bath application. *n* = 27 slices/4 macaques, *n* = 26 slices/9 mice. ***D***, Proportion of dopamine peak amplitudes blocked by the nicotinic receptor blocker DHβE in macaque (red) and mouse (blue) dorsal striatum from male (white) and female (orange) animals. Bars are mean ± SEM, and symbols are individual values. ***E***, Time course of dopamine peak amplitudes as 1 and 10 μM DHβE were bath-applied in macaque caudate and putamen slices. Data is mean ± SEM normalized to predrug application. ***F***, Time course of the dopamine peak amplitudes during application of nicotinic blocker conotoxin-PIA (0.1 µM) followed by 1 µM DHβE. Data is mean ± SEM normalized to predrug application for macaque (red) and mouse (blue), *n* = 5 slices/2 macaque; 15/2 mice.

We next examined the effects of pharmacological manipulations on evoked dopamine release recorded in dorsal striatum slices. We first examined the regulation by nicotinic acetylcholine (ACh) receptors (nAChR) by comparing evoked dopamine release levels before and after application of the β_2_ subunit containing nAChR blocker DHβE. Across species (Fig. 3*A–C*), evoked dopamine levels were significantly smaller in the presence of DHβE at the highest stimulation tested (main effect of blocker *F*_(1,33)_ = 118.2, *p* < 0.001). Pairwise comparisons found that both macaques (*F*_(1,52)_ = 5.9, *p* = 0.019) and mice (*F*_(1,14)_ = 16.3, *p* = 0.012) had significantly smaller dopamine responses following DHβE. There was also a significant interaction between species and drug condition (*F*_(1,33)_ = 31.6, *p* < 0.001), suggesting the effects of nAChR were different. Indeed, the magnitude of the DHβE suppression was smaller in macaques than in mice (41 ± 3 vs 64 ± 5% decrease in mice).

Calculating the fraction of evoked dopamine that was dependent versus independent of nAChR activity (Fig. 3*D*) revealed that the nAChR-dependent component was larger in mice than that in macaque, and correspondingly the nAChR-independent component was smaller in mice than that in monkeys (*t*_(39)_ = 6.8, *p* < 0.001). To determine whether this species difference reflects poor sensitivity to the receptor blocker in macaques, we carried out an additional experiment in which we increased the concentration of DHβE 10-fold. However, increasing the blocker concentration to 10 µM did not further reduce evoked dopamine levels relative to the 1 µM application (Fig. 3*E*; *t*_(3)_ = 1.14, *p* = 0.337). Thus, the 1 µM concentration of DHβE appears sufficient to block the existing nicotinic receptors in the macaques, but these β2-containing receptors contribute less to the overall evoked dopamine release triggered by electrical stimulation in macaques than in mice.

We further examined the subtype composition of nAChRs that contribute to dopamine release in mice and monkeys using another receptor blocker. α-Conotoxin-P1A (Ctx-PIA) is a blocker for nAChRs that contain α_6_ subunits (Dowell et al., 2003). Application of Ctx-PIA significantly reduced evoked dopamine signals in both mice (23 ± 1% reduction) and macaques (41 ± 1% reduction; Fig. 3*F*; main effect of drug *F*_(17,323)_ = 38.34, *p* < 0.0001). However, the effect of blocking α_6_-containing nicotinic receptors was larger in macaques (macaque vs mouse post hoc test, *t*_(15)_ = 2.76, *p* = 0.006). Subsequent addition of DHβE produced no additional decrease in macaque (6 ± 1% reduction, post hoc macaque Ctx-PIA vs DHβE *t*_(5) _= 0.6, *p* = 0.5) but further decreased dopamine signals in mice by 37% to 40 ± 3% from the predrug administration (post hoc mouse Ctx-PIA vs DHβE *t*_(15)_ = 8.4, *p* < 0.0001). There was a significant interaction between species and pharmacology when we compared Ctx-PIA and DHβE conditions (*F*_(17,323)_ = 3.4, *p* < 0.0001). This result indicates that α_6_-containing nAChRs exert the most control over dopamine release in the caudate and putamen of macaques and suggests a loss over the course of evolution of non-α_6_-containing receptors in macaques, potentially accounting for the diminished cholinergic-mediated dopamine release and the smaller evoked dopamine signals in monkey slices.

**Figure 4.**
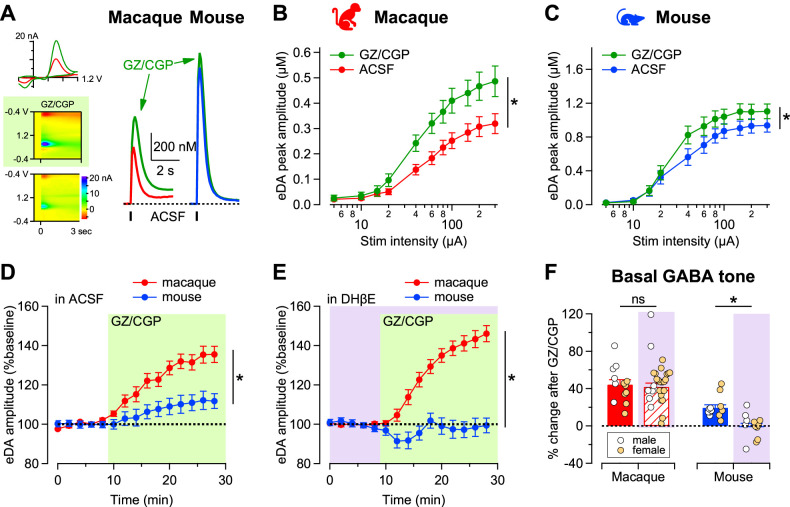
Stronger inhibitory modulation by GABA over striatal dopamine signals in macaques than mice. ***A***, Representative traces of dopamine signals (right), the current–voltage plots (left top), and the voltammograms (left middle and bottom) evoked by a single pulse of electrical stimulation (tick) in macaque (red) and mouse (blue) striatum before and after (green) bath application of the GABA receptor blockers (5 μM gabazine and 2 μM CGP55845). ***B***, ***C***, The input–output curves of dopamine amplitude evoked by stimulation of increasing intensity before and after (green) GABA receptor blockers in macaque (***B***, red) and mouse (***C***, blue). Symbols and lines are mean ± SEM. *n* = 13 slices/3 macaque; 12 slices/6 mice. ***D***, ***E***, Time course of the dopamine amplitudes evoked at 300 µA during application of the GABA receptor blockers when done in the (***D***) absence (*n* = 11 slices/4 macaque; 12 slices/6 mice) or (***E***) presence of 1 µM DHβE in macaque (red) and mouse (blue) striatum (*n* = 29 slices/4 macaque; 13 slices/4 mice). Amplitude is normalized to predrug application (***E***). Symbols and lines are mean ± SEM. ***F***, Percent change in the dopamine peak amplitudes evoked by 300 µA after GABA receptor blockers in macaque (red) and mouse (blue) bars were plotted as bar graphs together with individual values for the two species. Bars and lines are mean ± SEM, and symbols represent data from single experiments shown in white symbols for male and orange for female. *n* = 13 slices/4 macaques in ACSF and 31 slices/4 macaques in DHβE; 12 slices/6 mice in ACSF and 13 slices/4 mice in DHβE.

Over 95% of striatal neurons are GABAergic and the striatum is therefore dominated by inhibition. Thus, we next examined the effect of blocking GABA receptors on evoked dopamine release. We found that a combination of gabazine and CGP55845 (GZ/CGP), which block GABA_A_ and GABA_B_ receptors, respectively, increased evoked dopamine release (Fig. 4*A–C*). The voltammograms and current–voltage plots showed selective changes in the carbon fiber currents only at two voltages characteristic for dopamine oxidation and reduction, strongly suggesting an increase in the evoked extracellular dopamine concentration after blockers. The potentiation was seen in both species [Fig. 4*A–C*; main effect of blockers *F*_(2,48)_ = 35.86, *p* < 0.0001 and species (*F*_(1,21)_ = 14.42, *p* < 0.001)]; but it was proportionally larger in macaques than mice (Fig. 4*D*; main effect of species *F*_(1,21)_ = 14.42, *p* = 0.001). We found a significant species × GABA blockers interaction (*F*_(14,294)_ = 9, *p* < 0.0001), suggesting a stronger inhibitory tone in the primate striatum compared with mouse.

To examine possible interactions between GABAergic- and cholinergic-mediated modulation of dopamine release, we tested the effect of GABA blockers after application of the nicotinic receptor blocker DHβE (Fig. 4*E*). In the presence of DHβE, GABA receptor blockers still produced a substantial increase in dopamine release in macaque but not in mice (Fig. 4*E*; *t*_(39)_ = 6.8, *p* < 0.001). Blocking nicotinic receptors did not prevent the disinhibition produced by GABA receptor blockers in macaques, suggesting the mechanisms underlying the GABA-mediated modulation of release are independent from those of ACh and could be additive in the macaque. In mouse, the GABA blocker-mediated disinhibition was not observed after DHβE, suggesting the cholinergic and GABAergic modulation of dopamine release work, at least partially, through a common mechanism, in agreement with a recent report (Brill-Weil et al., 2024). However, gabazine has also been reported to decrease the sensitivity of carbon fibers to dopamine (Patel et al., 2024). In our experiments, using 5 µM gabazine led to an approximate 18 ± 10% decrease in dopamine sensitivity. In the presence of a nicotinic receptor blocker, gabazine induced a rapid suppression of evoked signals, followed by a recovery to baseline (Fig. 4*E*). This suggests that the initial suppression might be due to gabazine's effects on carbon fiber sensitivity, while the recovery to baseline could result from gabazine's effect on disinhibiting dopamine release.

To further assess the modulation and their interactions, we evaluated the relative proportion of GABA blocker potentiation in each species, before and after nAChR blockers (Fig. 4*F*). Evoked dopamine release was significantly greater across species when GABA blockers were applied without DHβE (main effect of DHβE *F*_(1,69)_ = 5.5, *p* = 0.021). As stated before, the potentiation of evoked dopamine responses by GABA blockers is larger in macaques than that in mice (*t* = 2.9, *p* = 0.005). Furthermore, in macaques GABA receptor blockers potentiated dopamine release similarly before and after DHβE (*t* = 1.1, *p* = 0.25), suggesting that inhibition is not acting on the nicotinic component of the release mechanism in macaques. In the mouse, however, the small potentiation by GABA receptor blockers is absent after DHβE (*t* = 2.1, *p* = 0.039), suggesting that inhibition affects the cholinergic-dependent dopamine release mechanism.

**Figure 5.**
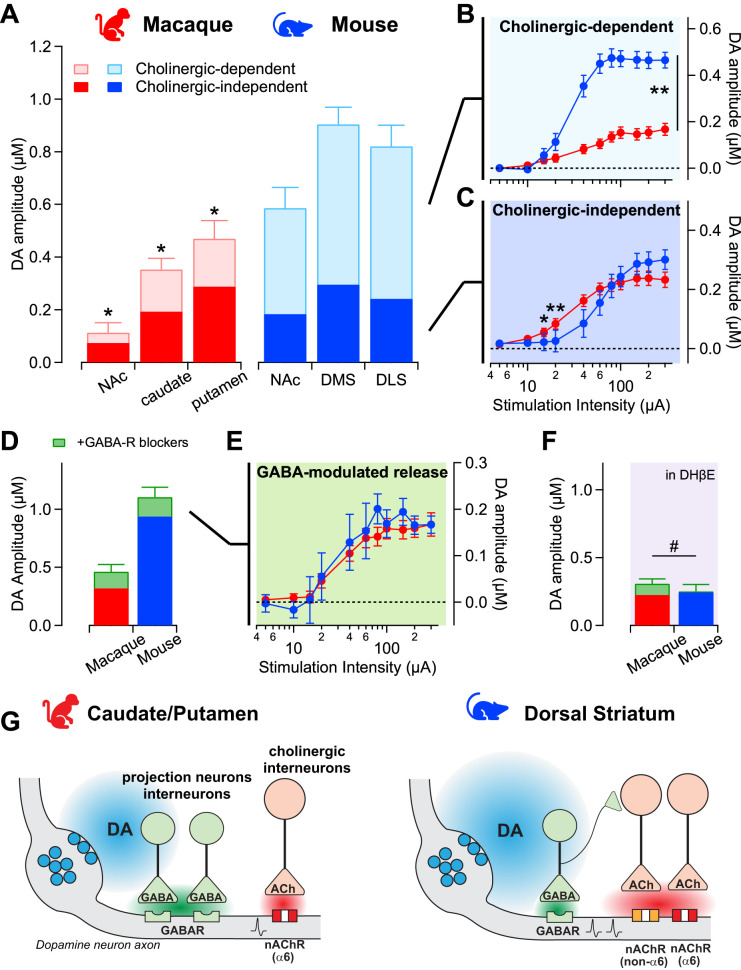
Shift from acetylcholine to GABA modulation of dopamine signals in macaque compared with mice. ***A***, Bar plot shows the overall magnitude of the evoked dopamine signals for maximal stimulation (300 µA) across striatal subregions in macaque (red) and mouse (blue). Solid portion represents the amplitude remaining after DHβE, denoting cholinergic-independent release. Light portion represents the amplitude of cholinergic-dependent release, which was blocked by DHβE. *n* = 61 slices/13 macaque; *n* = 61 slices/11 mice. * significant difference from mouse subregion. NAc, nucleus accumbens; DMS, dorsomedial striatum; DLS, dorsolateral striatum. ***B***, The input–output curves of the cholinergic-dependent dopamine signals in the macaque caudate (red) and the mouse DMS (blue). ***C***, The input–output curves of the non-ACh-dependent dopamine signals in the macaque caudate (red) and the mouse DMS (blue). *n* = 27 slices/4 macaques, *n* = 26 slices/9 mice. ***D***, Amplitude of evoked dopamine signals at maximal stimulation (300 µA) for macaque caudate (red) and mouse DMS (blue) before and after GABA receptor antagonists gabazine and CGP (green). * main effect of GABA blockers. *n* = 13 slices/3 macaque; 12 slices/6 mice. ***E***, The input–output curves of the GABA-modulated dopamine signals in the macaque caudate (red) and the mouse DMS (blue). *n* = 12–13 slices/3–4 animals. ***F***, Amplitude of evoked dopamine signals remaining after DHβE at maximal stimulation (at 300 µA) for macaque caudate (red) and mouse DMS (blue) before and after GABA receptor antagonists gabazine and CGP (green) in the presence of nicotinic receptor antagonist. ^#^ significant interaction species × GABA blocker. *n* = 31 slices/4 macaques; 13 slices/4 mice. For all plots, symbols and lines are mean ± SEM. ***G***, Diagram summarizing main findings and a model for interpreting the species differences.

Finally, we measured the overall magnitudes of the cholinergic- and GABAergic-mediated effects on regional evoked dopamine release in mice and macaque. We observed larger evoked dopamine signals in mice than macaques and regional differences in both species (Fig. 5*A*; main effect of region *F*_(2,116)_ = 8.59, *p* = 0.0003 and species *F*_(1,116)_ = 58.20, *p* < 0.0001; no interaction *p* = 0.32), in agreement with previous work (Calipari et al., 2012). Isolating the cholinergic-dependent dopamine release using pharmacology shows differences between striatal subregions and species (main effect of region *F*_(2,116)_ = 5.87, *p* = 0.0037 and species *F*_(1,116)_ = 95.96, *p* < 0.0001; no interaction *p* = 0.66). These cholinergic-dependent signals were smaller in macaque than those in mouse for all subregions (post hoc macaque vs mouse *t*'s > 4.3, *p*'s < 0.0001). We then analyzed and compared the input–output curves for the dorsomedial striatum/caudate, for which we have the most data points in both species. In the mouse, these cholinergic-dependent dopamine signals are twice the size of the remaining signals, and the input–output curve shows an upward shift with no change in sensitivity compared with the curve in macaques (Fig. 5*B*; significant interaction stimulation × species *F*_(10,510)_ = 6.57, *p* < 0.0001; intensity 15–20 μA mouse vs macaque *t* > 3.2, *p* < 0.01). On the contrary, the remaining evoked dopamine signal, which is independent of nicotinic receptors, has similar amplitudes in mice and macaques (Fig. 5*A*, no species effect *F*_(1,118)_ = 2.44, *p* = 0.12), and there are regional differences (*F*_(2,118)_ = 8.57, *p* = 0.0003). The input–output curve of this nicotinic-independent component in the macaques also showed a leftward shift compared with mice and an interaction between stimulation and species (Fig. 5*C*; *F*_(10,330)_ = 3.12, *p* = 0.0008), suggesting evoked dopamine signals in macaques have higher sensitivity to electrical stimulation.

Evaluation of the isolated GABA-modulated component of evoked dopamine release revealed that GABA blockers produced robust potentiation (Fig. 5*D*) and displayed overlapping input–output curves in macaques and mice (Fig. 5*E*). The maximal amplitude of the GABA-modulated dopamine signals and the sensitivity to stimulation are similar in macaques and mice (no effect of species *F*_(1,23)_ = 0.25, *p* = 0.62). However, there were two differences between the species. First, the potentiation is proportionally larger in macaques because they have smaller overall initial signals (44 ± 5% in macaques vs 19 ± 3% in mice, *t* = 2.93, *p* = 0.0046). Second, the potentiation persisted after blocking nicotinic receptors mainly in macaques (Figs. 4*E*,*F*, 5*F*; 36 ± 4% in macaques vs 1 ± 3% in mice, *t* = 5.06, *p* < 0.0001), which may reflect that GABA-mediated modulation is more robust in macaque than mice.

## Discussion

The goal of this study was to perform a detailed characterization of ex vivo evoked dopamine signals in the striatum of macaques and mice. By using the same recording equipment and experimental conditions, our experiments allowed for a systematic comparison between the two species. We observed differences in the overall magnitude of evoked dopamine signals between species and regional variations within species, confirming and validating previous findings (Calipari et al., 2012). Our work also provides mechanistic insight by demonstrating that cholinergic-dependent dopamine release is smaller in macaques compared with mice (~40 vs ~60%) and that the tonic inhibition by GABA is larger in macaque (44 vs 19%), both of which contribute to the difference in amplitude between species.

### Technical and other biological considerations on dopamine measurements

Dopamine concentration was measured using both the classical electrochemical method of FSCV and a more recent approach—photometry, which utilizes engineered dopamine receptors that increase fluorescence emission upon dopamine binding (Dong et al., 2022). Although each method comes with limitations, both techniques showed consistently lower concentrations of extracellular dopamine in macaques compared with mice. One of the limitations of the photometry measurement is that the expression level of dopamine sensors can be different between the two species, and it could affect the comparison. Lower expression level of the sensor in macaques could contribute to the disproportionately lower signals measured in macaque with photometry versus FSCV. Interestingly, in macaques, where evoked dopamine signals were smaller, the expression of dopamine sensors further reduced the dopamine concentration detected by the carbon fiber during FSCV. In mice, however, the expression of the dopamine sensors did not impact the dopamine concentration measured by FSCV.

A few other technical considerations are essential for interpreting these findings. Voltammetry has a slower detection rate compared with photometry (10 Hz vs 5 kHz) and lower sensitivity, as the carbon fiber electrode is inserted into the tissue and measures dopamine that “spills over” from release sites. In contrast, dopamine sensors like dLight1.3b and GRAB-DA1m have affinities similar to endogenous D_1_ and D_2_ receptors, respectively, and are expressed on cell membranes, allowing them to detect extracellular dopamine close to the release sites. However, due to their high ligand affinity, these sensors may exhibit a reduced effective dynamic range and potentially interfere with dopamine binding to endogenous receptors, thus affecting signaling. Our results indicate that in macaques, the expression of dopamine sensors diminished the dopamine concentration detected by FSCV, which may reflect a reduction in available extracellular dopamine for binding to endogenous receptors when the sensors are expressed. These findings highlight the importance of considering potential interference when using dopamine sensors for in vivo measurements during behavior.

The circadian influence on dopamine content and evoked striatal release should be taken into account, as it may impact each species differently. Since all experiments were conducted during the light phase of the circadian cycle, circadian variations in dopamine levels likely affect macaques and mice differently, given that mice are nocturnal. Enzymes involved in dopamine synthesis and degradation, such as monoamine oxidase A and tyrosine hydroxylase (TH), are regulated by clock genes and the circadian rhythm (Hampp et al., 2008; Sidor et al., 2015; Verwey et al., 2016). Based on these studies, we expect that TH expression and activity are at their lowest during the sleep/inactive phase of the mouse circadian cycle, which coincides with when they were tested. Therefore, while we cannot rule out the possibility that circadian factors contribute to the observed interspecies differences in evoked dopamine levels, these factors alone cannot explain the higher evoked levels observed in mice compared with macaques.

### Cholinergic modulation of dopamine release

Cholinergic modulation of dopamine release is observed across all striatal subregions; however, in both species, it is enhanced in the medial and lateral dorsal striatum and less pronounced in the NAc. After blocking the cholinergic-dependent signals, the remaining evoked dopamine signals are of similar amplitudes between species, with the macaque signals exhibiting higher sensitivity to stimulation while nAChRs are blocked. Additionally, in the mouse, the cholinergic component of evoked dopamine release is dependent on both α_6_- and non-α_6_-containing nAChRs. Whereas non-α6-containing nAChRs contribute up to half of evoked dopamine release in mice, α_6_-containing nAChRs are responsible for nearly all cholinergic-dependent dopamine release measured in vitro in the macaque caudate and putamen (Fig. 3*F*). Therefore, it is possible that there is an evolutionary loss of this non-α_6_-containing nAChR in macaque, or specific gain in the mice, that is responsible for the diminished overall cholinergic-dependent dopamine release in the macaque striatum. Alternative explanations for the interspecies difference include nAChR density, axonal location of the receptors, strength and dynamics of presynaptic ACh release, and the density of cholinergic terminals, among others. These factors should be investigated in future studies.

### GABA modulation of dopamine release

We also identified local modulation of dopamine release by the neurotransmitter GABA. GABA is abundant in the striatum, with over 95% of the cells releasing GABA (Burke et al., 2017). Furthermore, while most afferents to the striatum release glutamate, GABA release from midbrain dopamine neurons and midbrain GABAergic projection neurons has also been reported in mice (Tritsch et al., 2016). The local modulation by GABA, revealed by application of GABA_A_ and GABA_B_ receptor blockers, potentiated dopamine release in both macaques and mice. These findings agree with recent published work showing GABA_A_ receptor-mediated suppression of dopamine release in mice (Kramer et al., 2020; Brill-Weil et al., 2024; Patel et al., 2024).

The absolute magnitude of the dopamine potentiation in the presence of GABA blockers was similar in macaques and mice, with no change in the sensitivity to stimulation. But since the overall evoked release in macaques was one-third of that of mice, the potentiation was proportionally larger in macaques than mice (~50 vs ~15% in mice). Inhibition and disinhibition are common features of striatal networks, aiding in the processing and decoding of information within these GABA-dominated circuits. Dopamine itself has been shown to modulate GABA release in the striatum. This is thought to be a key action of dopamine, allowing this neuromodulator to regulate neuronal excitability and subsequently long-term plasticity in the striatum (Dobbs et al., 2016; Burke et al., 2017; Burke and Alvarez, 2022). Based on the findings of the current study, we speculate that local inhibition and disinhibition of dopamine release by GABA is another relevant striatal feature that is evolutionarily conserved and possibly expanded in primate basal ganglia.

### GABA–acetylcholine interaction

We found that GABA modulation of dopamine transmission persisted in the macaque striatum even after blocking cholinergic-mediated dopamine release. In mice, however, GABA-mediated modulation of dopamine release was reduced following the inhibition of cholinergic-mediated release. Given that GABA receptor inhibition is weaker in mice and that the GABA receptor blocker gabazine reduces carbon fiber sensitivity, we advise caution in interpreting these findings.

Additionally, in mice, GABA blockers potentiate evoked dopamine responses during burst stimulation, even after blocking cholinergic-mediated release (Lopes et al., 2019; Brill-Weil et al., 2024; Patel et al., 2024). This differential effect of GABA blockers, which becomes evident with longer stimulations, suggests that GABA blockers are more effective under conditions where axonal depolarization is integrated over time. Alternatively, the prolong stimulation may reveal the autoinhibition by GABA corelease from the same dopamine terminals (Patel et al., 2024). The present results indicate that the regulation or autoregulation by GABA receptors also occurs in macaque striatum, and they suggest it may be even more influential than in mouse.

Despite these caveats, there is a clear interspecies difference in the magnitude of GABA receptor-mediated disinhibition that cannot be explained solely by changes in dopamine sensitivity. We speculate that this difference between macaques and mice may reflect a stronger GABA tone and/or greater integration of axonal depolarization in macaques. Alternatively, macaques may have an expanded expression of GABA receptors on dopamine axons, and/or an increased number or strength of axo-axonic interactions.

### Future directions

Future efforts will focus on expanding the understanding of the developmental changes in dopamine release capacity and modulation throughout the lifespan of macaque and mice. Numerous studies have shown that the dopaminergic system undergoes significant transformations during adolescence (Avramescu et al., 2024), leading us to expect age-related differences in dopamine release properties within each species. While the current study was conducted on adult animals of both sexes, we acknowledge two key limitations: our inability to better match the age range across species and the lack of a sufficient number of animals to address sex differences with adequate statistical power. Our ongoing developmental study aims to overcome these limitations and enhance our understanding of sex- and age-related changes in dopamine release and modulation over the lifespan in both mice and macaques.

Another key goal in our ongoing work with macaques is to establish reliable in vivo measurements of dopamine using fluorescent sensors. The primary challenge we face is ensuring consistent sensor expression. Our current ex vivo experiments are invaluable in addressing this issue, as they allow us to evaluate the effectiveness of various viral vectors, enhancers, incubation times, and sensor types. Successfully achieving reliable in vivo dopamine measurements in macaques will significantly advance our ability to explore the functional implications of the differences in dopamine release and regulation that we have identified between macaques and mice.

In summary, we found several differences in the regulation of evoked dopamine release between the species. There was a stronger cholinergic contribution in mice, and two subtypes of nicotinic receptors are involved. In the macaque, only one subtype of nicotinic receptor is involved, and the cholinergic contribution is smaller across dorsostriatal subregions. The GABA-mediated inhibition of dopamine release is proportionally stronger in macaques, and it is independent of the cholinergic modulation. The study broadens our understanding of striatal dopamine release properties and regulation in primates.

## Data Availability

This study did not generate new unique reagents. The datasets supporting the current study have been deposited at Mendeley.com (https://doi.org/10.17632/hgd3mkypwj.1) and are publicly available as of the date of publication. No original code was generated. Any additional information required to reanalyze the data reported in this paper is available from the lead contact upon request.
